# Association between the atherogenic index of plasma and left ventricular hypertrophy in patients with obstructive sleep apnea: a retrospective cross-sectional study

**DOI:** 10.1186/s12944-024-02170-5

**Published:** 2024-06-12

**Authors:** Min Sun, Chao Liang, Hui Lin, Zhiyan Chen, Meng Wang, Shijie Fang, Tian Tian, Yujing Yang, Qunzhong Tang, Erming Zhang, Qiang Tang

**Affiliations:** 1 Department of Cardiology, Peking University Shougang Hospital, Beijing, China; 2 Heart Center, The First Hospital of Hebei Medical University, Shijiazhuang, Hebei China; 3 Department of Respiratory, Peking University Shougang Hospital, Beijing, China

**Keywords:** Atherogenic index of plasma, Left ventricular hypertrophy, Obstructive sleep apnea, Insulin resistance

## Abstract

**Background:**

The atherogenic index of plasma (AIP) is a simple and reliable marker of insulin resistance and is closely associated with various cardiovascular diseases (CVDs). However, the relationships between AIP and left ventricular (LV) geometric indicators have not been adequately assessed. This study was carried out to investigate the association between AIP and LV geometric abnormalities in obstructive sleep apnea (OSA) patients.

**Methods:**

This retrospective cross-sectional study included a total of 618 OSA patients (57.3 ± 12.4 years, 73.1% males, BMI 28.1 ± 4.2 kg/m^2^) who underwent echocardiography. Patients with OSA were diagnosed with clinical symptoms and an apnea-hypopnea index ≥ 5.0. LV hypertrophy (LVH) was defined as left ventricular mass index (LVMI_h2.7_) ≥ 50.0 g/m^2.7^ for men and 47.0 g/m^2.7^ for women. AIP was calculated as log_10_ (TG/HDL-C).

**Results:**

Compared with the non-LVH group, AIP was significantly higher in the LVH group (0.19 ± 0.29 vs 0.24 ± 0.28, *P* = 0.024) and the concentric LVH group (0.18 ± 0.29, 0.19 ± 0.30, 0.20 ± 0.26 and 0.29 ± 0.29 in the control, concentric remodeling, eccentric hypertrophy and concentric hypertrophy groups, respectively, *P* = 0.021). Meanwhile, in the group of patients with the highest AIP tertile, the levels of LVMI_h2.7_ (42.8 ± 10.5, 43.2 ± 9.3 and 46.1 ± 12.1 in the T1, T2 and T3 groups, respectively, *P* = 0.003), and the prevalence of LVH (25.2%, 24.0% and 34.6% in the T1, T2 and T3 groups, respectively, *P* = 0.032) and concentric LVH (10.7%, 9.8% and 20.2% in the T1, T2 and T3 groups, respectively, *P* = 0.053) were higher compared with those in the other groups. Positive correlations between AIP and LV geometric indicators including the LVMI_h2.7_, LVMI_BSA_, LV mass (LVM), diastolic left ventricular inner diameter (LVIDd), diastolic left ventricular posterior wall thickness (PWTd) and diastolic interventricular septal thickness (IVSTd), were revealed according to correlation analysis (*P* < 0.05). Furthermore, AIP was independently associated with LVMI_h2.7_ according to multivariate linear regression model (β = 0.125, *P* = 0.001). Notably, AIP remained independently associated with an elevated risk of LVH [odds ratio (OR) = 1.317 per 1 standard deviation (SD) increment, 95% confidence interval (CI): 1.058 - 1.639, *P* = 0.014) and concentric LVH (OR = 1.545 per 1 SD increment, 95% CI: 1.173 - 2.035, *P* = 0.002) after fully adjusting for all confounding risk factors by multivariate logistic regression analyses.

**Conclusions:**

AIP was independently associated with an increased risk of LVH and concentric LVH in OSA patients. Therefore, AIP, as a practical and cost-effective test, might be useful in monitoring hypertrophic remodeling of the heart and improving CVDs risk stratification in clinical management of OSA.

## Background

Obstructive sleep apnea (OSA), characterized by recurrent partial or total obstructions of the upper airway during sleep, is a well-known public health problem that affects more than 10% of the general population, primarily overweight and obese patients [1]. Accumulating studies have shown that OSA is associated with an increased risk of cardiovascular morbidity and mortality [2]. The main myocardial structural remodeling observed in OSA patients is left ventricular hypertrophy (LVH), a major independent risk factor for cardiovascular events [3, 4]. Despite the growing awareness of the need to clinically identify LVH in OSA patients for cardiovascular diseases (CVDs) risk stratification, there is still a lack of convenient and practical parameters to supplement and improve LVH detection in clinical practice.

Previous studies have shown that insulin resistance (IR) increases with the severity of OSA and that IR is an important factor that accelerates LVH by stimulating cardiomyocyte hypertrophy, oxidative stress and inflammation [5–7]. Recently, the atherogenic index of plasma (AIP), determined with the formula log_10_ [triglyceride (TG)/high-density lipoprotein cholesterol (HDL-C)], was shown to serve as a convenient and reliable marker of IR and to better reflect the pathogenicity and specificity of dyslipidemia than high TG or low HDL-C levels alone [8–10]. AIP is closely associated with various CVDs, such as coronary artery disease (CAD), coronary artery calcification, in-stent restenosis, poor collateral formation of coronary artery total occlusion, hypertension and mitral annular calcification [11–16]. Several studies have investigated the relationship between TG/HDL-C ratio and LVH in obese children and the general population [17–21]. TG/HDL-C ratio was found to positively correlate with left ventricular mass index (LVMI), and a high TG/HDL-C ratio was demonstrated to be independently associated with an increased risk of concentric LVH [19–21]. However, no prior studies have explored the clinical value of AIP for evaluating LVH in patients with OSA. Therefore, the purpose of this study was to explore the association between AIP and LVH in OSA patients, so as to provide a possible reference for early detecting and monitoring cardiac adverse remodeling in patients with OSA.

## Methods

### Study population

Consecutive patients who were diagnosed with OSA and underwent echocardiography at the cardiovascular department of Peking University Shougang Hospital from February 2016 to August 2022 were retrospectively enrolled. The diagnosis of OSA was made based on clinical symptoms of OSA (snoring, snorting, gasping, breathing pauses during sleep, daytime sleepiness or fatigue despite sufficient sleep) and an apnea-hypopnea index (AHI) ≥ 5.0 of the complete out of center sleep test (OCST) records. The study complied with the principles outlined in the Declaration of Helsinki and was approved by the local ethics committee of Peking University Shougang Hospital (IRBK-2023-017-01). Due to the retrospective design of the study, written consent from the patients could not be obtained.

Patients with central sleep apnea, previous treatment for OSA, hypoxemic lung disease (such as chronic obstructive pulmonary disease, interstitial lung disease, asthma, pulmonary embolism), congestive heart failure, old myocardial infarction, acute coronary syndrome, known atrial fibrillation, significant aortic or mitral valve diseases, hypertrophic cardiomyopathy, malignancy, infection, autoimmune disease, liver or kidney disease, thyroid disease, alcohol abuse or poor image quality were excluded from the study.

### Collection of demographic, medical and laboratory data

Demographic characteristics and medical data, including age, sex, systolic blood pressure (SBP), diastolic blood pressure (DBP), height, weight, history of hypertension, diabetes, CAD, antihypertensive medications, antihyperlipidemic medications, smoking and alcohol consumption, were obtained from medical records. Body mass index (BMI) was calculated as weight/height squared (kg/m^2^). Overweight was defined as 24.0 kg/m^2^ ≤ BMI < 28.0 kg/m^2^ and obesity was defined as BMI ≥ 28 kg/m^2^ [22]. Hypertension severity was divided into 2 stages according to blood pressure (BP) levels or medication use: stage-1, BP < 160/100 mmHg or BP under control with 1 or 2 antihypertensive drugs; stage-2, BP ≥ 160/100 mmHg or BP under control with ≥ 3 antihypertensive drugs [23, 24]. Laboratory data, including fasting blood glucose (FBG), TG, total cholesterol (TC), HDL-C and low-density lipoprotein cholesterol (LDL-C), were measured from fasting blood samples with AU5811 automatic biochemical analyser (Beckman Coulter, USA). AIP was calculated as log_10_ [TG (mmol/L)/HDL-C (mmol/L)]. Patients were divided into 3 groups according to the three tertiles of AIP: the T1 group (AIP < 0.07), the T2 group (0.07 ≤ AIP < 0.31) and the T3 group (AIP ≥ 0.31).

### OCST evaluation

Patients with clinically suspected OSA underwent OCST (Apnea Link Air, ResMed Germany Inc., Germany) after admission to the hospital. As described in a previous study [25], according to the American Association of Sleep Medicine (AASM) criteria, apnea was defined as a decrease to 0 - 20% of oronasal air flow for longer than 10 s; hypopnea was defined as a decrease in oronasal air flow by 50% for longer than 10 s or a decrease in both oronasal air flow by at least 30% and oxygen saturation by 4% for longer than 10 s. The apnea and hypopnea counts per hour were recorded as the AHI [26]. Parameters including the AHI, percentage of sleep duration with oxygen saturation < 90% (TS90), lowest pulse oxygen saturation (LSpO_2_) and mean oxygen saturation (SpO_2_) were recorded. OSA severity was divided into mild (AHI: 5.0 - 14.9) and moderate to severe (AHI ≥ 15.0) OSA according to the AHI.

### Echocardiographic examination

All patients underwent a transthoracic echocardiogram (GE Healthcare, Vivid S6 system, M4S-RS probe) at the cardiovascular department of Peking University Shougang Hospital. Echocardiographic parameters relevant to the current analysis, including left atrium diameter (LAD), diastolic left ventricular inner diameter (LVIDd), diastolic left ventricular posterior wall thickness (PWTd), diastolic interventricular septal thickness (IVSTd) and left ventricular ejection fraction (LVEF), were collected. Left ventricular mass (LVM) was calculated as follows: LVM (g) = 0.8*1.04*((IVSTd + PWTd + LVIDd)^3^ - LVIDd^3^) + 0.6. Body surface area (BSA) was calculated as follows: BSA (m^2^) = (weight [kg]^0.425^ * height [cm]^0.725^) * 0.007184. LVMI was calculated by dividing LVM by BSA according to the recommendation of the American Society of Echocardiography/European Association of Cardiovascular Imaging (ASE/EACVI) guidelines [27]. LVMI_h2.7_ was calculated by dividing LVM by height^2.7^. Considering the high prevalence of overweight and obesity in OSA patients, LVH was defined as LVMI_h2.7_ > 50.0 g/m^2.7^ for men or 47.0 g/m^2.7^ for women to avoid underdiagnosis of LVH according to the EACVI recommendation [27]. Relative wall thickness (RWT) was calculated as 2*PWTd/LVIDd. LV geometric patterns were classified as normal (RWT ≤ 0.42 and no LVH), concentric remodeling (RWT > 0.42 and no LVH), eccentric hypertrophy (RWT ≤ 0.42 and LVH), and concentric hypertrophy (RWT > 0.42 and LVH).

### Statistical analysis

Data management and statistical analysis were performed using SPSS 22.0 (IBM SPSS Statistics for Windows, USA). Histograms, probability plots and Kolmogorov-Smirnov test were performed to investigate the normality of the distributions of continuous variables. Normally distributed data are expressed as mean ± standard deviation (SD), and nonnormally distributed data are expressed as median (interquartile range). Student’s unpaired t-test and one-way analysis of variance (ANOVA) test were used for normally distributed data, while Mann-Whitney U test and Kruskal-Wallis test were used for nonnormally distributed data. Meanwhile, categorical variables were expressed as number (percentage). Comparison of categorical variables was analysed by chi square test. Pearson correlation test and Spearman’s rank correlation test were performed to evaluate the correlation between AIP and left ventricular (LV) geometric indicators. The association between AIP and LVMI was analysed with univariate and multivariate linear regression analysis. Variables which could be associated with LVMI and/or showed *P* < 0.1 in univariate analysis such as age, gender, BMI, SBP, diabetes mellitus, CAD, cigarette smoking, alcohol consumption, antihyperlipidemic medications and AHI were included in the multivariate analysis. Clinical, laboratory and OCST parameters that showed significant correlations were not included in the same regression model to avoid multicollinearity. The Standardized β coefficient for independent association of per 1 SD increase in AIP with LVMI were reported. Risk factors of LVH and concentric LVH were analysed with univariate and multivariate logistic regression analysis. Variables which could be associated with LVH and concentric LVH and/or showed *P* value < 0.1 in univariate analysis such as age, gender, BMI, cigarette smoking, alcohol consumption, diabetes mellitus, CAD, hypertension severity, OSA severity and antihyperlipidemic medications were included in the multivariate analysis. Clinical, laboratory and OCST parameters that showed significant correlations were not included in the same regression model to avoid multicollinearity. The odds ratios (ORs) and 95% confidence intervals (CIs) for independent association of per 1 SD increase in AIP with LVH and concentric LVH were reported. In addition, individuals were stratified into tertiles in accordance with the distribution of AIP to further evaluate the association between AIP and the risk of LVH and concentric LVH. The goodness-of-fit assumption was tested by the Hosmer-Lemeshow method and satisfied if *P* value > 0.05. A two-sided *P* value < 0.05 was considered to indicate statistical significance.

## Results

### Baseline characteristics

Initially, 684 OSA participants were enrolled, but 66 participants were excluded from the study because 14 participants lacked TG or HDL-C data, 20 participants lacked complete echocardiographic data, and 32 participants met the exclusion criteria, such as overt heart failure, valvulopathy, chronic atrial fibrillation, acute coronary syndrome, etc. Finally, a total of 618 patients were included in the study (452 males, aged 57.3 ± 12.4 years, averaged BMI 28.1 ± 4.2 kg/m^2^, 85.6% overweight and obesity), including 173 patients with LVH (28.0%) and 445 patients without LVH (72.0%). The distribution of AIP was normal, and the mean AIP of the study population was 0.20 ± 0.29.

### Clinical characteristics of patients by LVH and LV geometric patterns

**Fig. 1:**
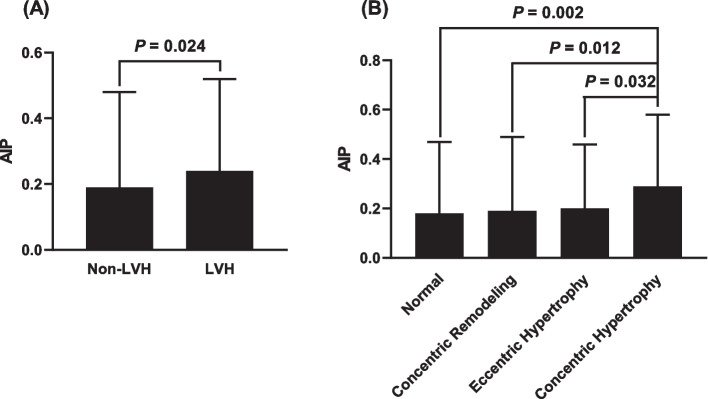
Comparisons of AIP between the non-LVH and LVH groups (**A**) and between different LV geometric patterns (**B**). AIP, atherogenic index of plasma; LVH, left ventricular hypertrophy; LV, left ventricular

Table 1 shows the clinical characteristics of patients by LVH. Patients with LVH were older and more likely to be women, had higher SBP, BMI and TG levels, and had lower HDL-C levels. Moreover, the prevalence of obesity, hypertension, use of ≥ 3 classes of antihypertensive medications and stage-2 hypertension were higher in patients with LVH. With regard to the OCST parameters, AHI and TS90 and the prevalence of moderate to severe OSA were higher while the mean SpO_2_ was lower in patients with LVH than in those without LVH (Table 1). In particular, the mean AIP was significantly increased in the LVH group (0.19 ± 0.29 vs 0.24 ± 0.28, *P* = 0.024, Fig. 1A) compared with those in the non-LVH group.

**Table 1:** Baseline characteristics of patients by LVH

Characteristics	Non-LVH (*n* = 445)	LVH (*n* = 173)	*P* value
Clinical parameters			
Age (years)	56.3 ± 12.3	60.0 ± 12.3	0.001^*^
Male gender, n (%)	341 (76.6)	111 (64.2)	0.002^*^
SBP (mmHg)	141 ± 19	150 ± 22	< 0.001^*^
DBP (mmHg)	84 ± 15	87 ± 18	0.136
Height (cm)	170 ± 8	165 ± 8	< 0.001^*^
Weight (kg)	78.9 ± 13.3	82.7 ± 15.3	0.003^*^
BMI (kg/m^2^)	27.3 ± 3.8	30.2 ± 4.4	< 0.001^*^
Obesity, n (%)	172 (38.7)	123 (71.1)	< 0.001^*^
Alcohol consumption, n (%)	109 (24.5)	33 (19.1)	0.151
Cigarette smoking, n (%)	185 (41.6)	60 (34.7)	0.116
Hypertension, n(%)	331 (74.4)	160 (92.5)	< 0.001^*^
Diabetes mellitus, n (%)	131 (29.4)	59 (34.1)	0.259
CAD, n (%)	198 (44.5)	89 (51.4)	0.120
Antihyperlipidemic medications, n (%)	105 (23.6)	46 (26.6)	0.437
≥ 3 classes of anti-hypertensive medications, n (%)	111 (24.9)	75 (43.4)	< 0.001^*^
Hypertension severity			
Stage-2 hypertension, n (%)	173 (38.9)	92 (53.2)	0.001^*^
Laboratory parameters			
FBG (mmol/L)	5.64 (5.11 - 6.80)	5.76 (5.20 - 7.20)	0.052
TG (mmol/L)	1.51 (1.08 - 2.23)	1.74 (1.17 - 2.40)	0.043^*^
TC (mmol/L)	4.47 ± 1.12	4.36 ± 1.02	0.275
HDL-C (mmol/L)	1.06 ± 0.27	1.01 ± 0.21	0.017^*^
LDL-C (mmol/L)	2.62 ± 0.82	2.56 ± 0.74	0.373
AIP	0.19 ± 0.29	0.24 ± 0.28	0.024^*^
OCST parameters			
AHI (events/h)	17.4 (10.0 - 33.7)	20.4 (11.6 - 36.4)	0.030^*^
Mean SpO_2_ (%)	94 (93 - 95)	93 (92 - 95)	< 0.001^*^
LSpO_2_ (%)	80 (74 - 83)	79 (73 - 82)	0.081
TS90 (%)	23 (7 - 54)	40 (11 - 99)	< 0.001^*^
OSA severity			0.035^*^
Mild OSA	193 (43.4)	59 (34.1)	
Moderate to severe OSA	252 (56.6)	114 (65.9)	
Echocardiographic parameters			
LVIDd (mm)	47 ± 4	51 ± 4	< 0.001^*^
PWTd (mm)	10 (9 - 11)	12 (11 - 13)	< 0.001^*^
IVSd (mm)	9 (9 - 10)	10 (10 - 11)	< 0.001^*^
LAD (mm)	36 ± 4	39 ± 6	< 0.001^*^
LVEF (%)	65 ± 5	63 ± 6	< 0.001^*^
LVM (g)	162.3 ± 29.3	224.7 ± 47.4	< 0.001^*^
LVMI (g/m^2^)	85.5 ± 12.9	118.1 ± 19.7	< 0.001^*^
LVMI_h2.7_ (g/m^2.7^)	38.8 ± 6.1	57.5 ± 8.5	< 0.001^*^
RWT	0.40 ± 0.05	0.43 ± 0.06	< 0.001^*^

*Abbreviations*: *LVH* left ventricular hypertrophy, *SBP* systolic blood pressure, *DBP* diastolic blood pressure, *BMI* body mass index, *CAD* coronary artery disease, *FBG* fasting blood glucose, *TG* triglyceride, *TC* total cholesterol, *HDL-C* high-density lipoprotein cholesterol, *LDL-C* low-density lipoprotein cholesterol, *AIP* atherogenic index of plasma, *OCST* complete out of center sleep test, *AHI* apnea–hypopnea index, *SpO*_*2*_ oxygen saturation, *LSpO*_*2*_ lowest pulse oxygen saturation, *TS90* percentage of sleep duration with oxygen saturation (SpO_2_) < 90%, *OSA* obstructive sleep apnea, *LVIDd* diastolic left ventricular inner diameter, *PWTd* diastolic left ventricular posterior wall thickness, *IVSd* diastolic interventricular septal thickness, *LAD* left atrium diameter, *LVEF* left ventricular ejection fraction, *LVM* left ventricular mass, *LVMI* left ventricular mass index, *LVMI*_*h2.7*_ dividing LVM by height^2.7^, *RWT* relative wall thickness

^*^P value < 0.05

Then, the LVH group was further categorized into the concentric remodeling, eccentric hypertrophy and concentric hypertrophy groups. As shown in Table 2, among all 4 groups, patients in the concentric hypertrophy group achieved the highest SBP, DBP, BMI and TG and the highest percentage of female patients, obesity, hypertension, use of ≥ 3 classes of antihypertensive medications, stage-2 hypertension and moderate to severe OSA. Moreover, compared with those in the control, concentric remodeling and eccentric hypertrophy groups, AIP was significantly increased in the concentric hypertrophy group (*P* = 0.021, Fig. 1B).

**Table 2:** Baseline characteristics of patients by LV geometric pattern

	Normal	Concentric remodeling	Eccentric hypertrophy	Concentric hypertrophy	*P* value
Characteristics	(*n* = 307)	(*n* = 138)	(*n* = 89)	(*n* = 84)	
Clinical parameters					
Age (years)	56.4 ± 12.6	56.1 ± 11.5	60.9 ± 11.3^†#^	59.0 ± 13.3	0.007^*^
Male gender, n (%)	233 (75.9)	108 (78.3)	63 (70.8)	48 (57.1)	0.003^*^
SBP (mmHg)	140 ± 19	143 ± 20	146 ± 21^†^	154 ± 22^†#§^	< 0.001^*^
DBP (mmHg)	84 ± 15	86 ± 15	84 ± 18	89 ± 17	0.022^*^
Height (cm)	170 ± 8	170 ± 7	166 ± 7^†#^	164 ± 9^†#§^	< 0.001^*^
Weight (kg)	78.9 ± 13.6	78.9 ± 12.6	82.7 ± 14.3^†#^	82.7 ± 16.3^†#^	0.029^*^
BMI (kg/m^2^)	27.3 ± 3.8	27.3 ± 3.9	29.8 ± 4.3^†#^	30.6 ± 4.5^†#^	< 0.001^*^
Obesity, n (%)	117 (38.1)	55 (39.9)	61 (68.5)	62 (73.8)	< 0.001^*^
Alcohol consumption, n (%)	73 (23.8)	36 (26.1)	20 (22.5)	13 (15.5)	0.314
Cigarette smoking, n (%)	119 (38.8)	66 (47.8)	32 (36.0)	28 (33.3)	0.118
Hypertension, n(%)	219 (71.3)	112 (81.2)	78 (87.6)	82 (97.6)	< 0.001^*^
Diabetes mellitus, n (%)	83 (27.0)	48 (34.8)	31 (34.8)	28 (33.3)	0.261
CAD, n (%)	133 (43.3)	65 (47.1)	52 (58.4)	37 (44)	0.087
Antihyperlipidemic medications, n (%)	76 (24.8)	29 (21.0)	25 (28.1)	21 (25.0)	0.671
≥ 3 classes of anti-hypertensive medications, n (%)	66 (21.5)	45 (32.6)	27 (30.3)	48 (57.1)	< 0.001^*^
Hypertension severity					
Stage-2 hypertension, n (%)	104 (33.9)	69 (50.0)	37 (41.6)	55 (65.5)	< 0.001^*^
Laboratory parameters					
FBG (mmol/L)	5.57 (5.04 - 6.63)	5.82 (5.17 - 6.96)	5.79 (5.22 - 6.77)	5.75 (5.20 - 7.50)	0.033^*^
TG (mmol/L)	1.52 (1.08 - 2.18)	1.48 (1.08 - 2.30)	1.56 (1.09 - 2.12)	1.89 (1.28 - 2.61)^†^	0.030^*^
TC (mmol/L)	4.45 ± 1.09	4.52 ± 1.19	4.22 ± 1.00	4.51 ± 1.03	0.203
HDL-C (mmol/L)	1.06 ± 0.28	1.05 ± 0.23	1.03 ± 0.21	0.99 ± 0.21	0.126
LDL-C (mmol/L)	2.60 ± 0.83	2.66 ± 0.80	2.46 ± 0.73	2.66 ± 0.73	0.278
AIP	0.18 ± 0.29	0.19 ± 0.30	0.20 ± 0.26	0.29 ± 0.29^†#§^	0.021^*^
OCST parameters					
AHI (events/h)	16.0 (9.4 - 30.9)	21.9 (11.3 - 38.9)^†^	20.9 (10.4 - 35.4)	20.4 (11.8 - 38.4)	0.007^*^
Mean SpO_2_ (%)	94 (93 - 95)	94 (93 - 95)	92 (93 - 95)^†#^	92 (93 - 95)	0.003^*^
LSpO_2_ (%)	80 (75 - 83)	79 (73 - 83)	79 (72 - 82)	79 (73 - 82)	0.324
TS90 (%)	23 (7 - 55)	25 (7 - 51)	40 (13 - 112)^†#^	38 (11 - 98)	0.002^*^
OSA severity					0.025^*^
Mild OSA	143 (46.6)	50 (36.2)	33 (37.1)	26 (31.0)	
Moderate to severe OSA	164 (53.4)	88 (63.8)	56 (62.9)	58 (69.0)	
Echocardiographic parameters					
LVIDd (mm)	48 ± 3	44 ± 3^†^	53 ± 4^†#^	48 ± 4^#§^	< 0.001^*^
PWTd (mm)	9 (9 - 10)	10 (10 - 11)^†^	10 (10 - 11)^†^	11 (10 - 12)^†#§^	0.001^*^
IVSTd (mm)	10 (9 - 11)	11 (10 - 12)^†^	11 (10 - 13)^†#^	12 (12 - 14)^†#§^	< 0.001^*^
LAD (mm)	36 ± 3	36 ± 4	39 ± 4^†#^	40 ± 8^†#^	< 0.001^*^
LVEF (%)	65 ± 5	66 ± 5	61 ± 7^†#^	65 ± 6^§^	< 0.001^*^
LVM (g)	161.7 ± 29.6	163.8 ± 28.5	224.5 ± 43.1^†#^	224.9 ± 51.9^†#^	< 0.001^*^
LVMI (g/m^2^)	85.0 ± 12.6	86.4 ± 13.4	117.5 ± 19.0^†#^	118.7 ± 20.5^†#^	< 0.001^*^
LVMI_h2.7_ (g/m^2.7^)	38.6 ± 6.1	39.2 ± 6.0	56.4 ± 7.8^†#^	58.7 ± 9.1^†#^	< 0.001^*^
RWT	0.38 ± 0.03	0.46 ± 0.04^†^	0.38 ± 0.03^#^	0.48 ± 0.05^†§^	< 0.001^*^

*Abbreviations*: *LV* left ventricular, *SBP* systolic blood pressure, *DBP* diastolic blood pressure, *BMI* body mass index, *CAD* coronary artery disease, *FBG* fasting blood glucose, *TG* triglyceride, *TC* total cholesterol, *HDL-C* high-density lipoprotein cholesterol, *LDL-C* low-density lipoprotein cholesterol, *OCST* complete out of center sleep test, *AHI* apnea–hypopnea index, *SpO*_*2*_ oxygen saturation, *LSpO*_*2*_ lowest pulse oxygen saturation, *TS90* percentage of sleep duration with oxygen saturation < 90%, *OSA* obstructive sleep apnea, *LVIDd* diastolic left ventricular inner diameter, *PWTd* diastolic left ventricular posterior wall thickness, *IVSd* diastolic interventricular septal thickness, *LAD* left atrium diameter, *LVEF* left ventricular ejection fraction, *LVM* left ventricular mass, *LVMI* left ventricular mass index, *LVMI*_*h2.7*_ dividing LVM by height^2.7^, *RWT* relative wall thickness

^*^P value < 0.05

^†^ vs. Normal, *P* value < 0.05

^#^ vs. Concentric remodeling, *P* value < 0.05

^§^ vs. Eccentric hypertrophy, *P* value < 0.05

### Clinical characteristics and echocardiography data of patients by AIP

AIP was calculated for each patient, and the study population was divided into 3 groups: the T1 group (AIP < 0.07, *n* = 206), T2 group (0.07 ≤ AIP index < 0.31, *n* = 204) and T3 group (AIP index ≥ 0.31, *n* = 208).

**Fig. 2:**
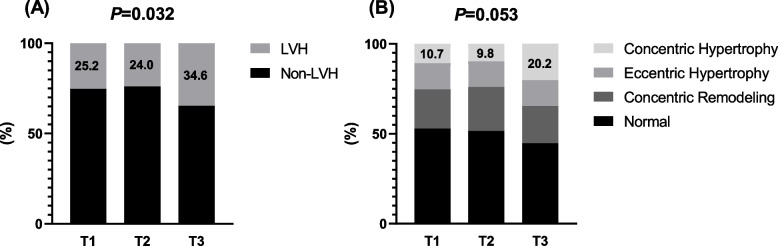
Comparisons of the percentages of patients with LVH (**A**) and concentric LVH (**B**) according to tertiles of AIP. LVH, left ventricular hypertrophy; AIP, atherogenic index of plasma; T1, AIP < 0.07; T2, 0.07 ≤ AIP < 0.31; T3, AIP ≥ 0.31

As demonstrated in Table 2, compared with patients in the T1 and T2 groups, those in the T3 group were younger and were more likely to be men and had higher DBP, BMI, FBG, TG, TC, and LDL-C levels and lower HDL-C levels. Moreover, the prevalence of obesity, cigarette smoking, use of ≥ 3 classes of antihypertensive medications and stage-2 hypertension were higher in the T3 group than those in the T1 and T2 groups. With regard to the echocardiography parameters, a significant difference was observed in LVIDd, PWTd, IVSd, LVM, LVMI, LVMI_h2.7_ and RWT, with greater values in the T3 group (Table 3). In particular, compared with those in the T1 and T2 groups, the mean LVMI_h2.7_ level was significantly increased in the T3 group (42.8 ± 10.5, 43.2 ± 9.3 and 46.1 ± 12.1 in the T1, T2 and T3 groups, respectively, *P* = 0.003, Table 3). Moreover, the prevalence of LVH (25.2%, 24.0% and 34.6% in the T1, T2 and T3 groups, respectively, *P* = 0.032, Fig. 2A) and concentric LVH (10.7%, 9.8% and 20.2% in the T1, T2 and T3 groups, respectively, *P* = 0.053, Fig. 2B) were higher in the T3 group than those in the T1 and T2 groups.

**Table 3:** Clinical characteristics and echocardiography data of patients by AIP

	T1 (AIP < 0.07)	T2 (0.07 ≤ AIP < 0.31)	T3 (AIP ≥ 0.31)	*P* value
Characteristics	(*n* = 206)	(*n* = 204)	(*n* = 208)	
Clinical parameters				
Age (years)	61.8 ± 11.3	57.0 ± 11.8^†^	53.2 ± 12.5^†#^	< 0.001^*^
Male gender, n (%)	138 (67.0)	149 (73.0)	165 (79.3)	0.018^*^
SBP (mmHg)	141 ± 20	144 ± 22	145 ± 20	0.053
DBP (mmHg)	81 ± 14	86 ± 17	88 ± 16^†^	< 0.001^*^
Height (cm)	167 ± 8	169 ± 8^†^	169 ± 9^†^	0.018^*^
Weight (kg)	75.5 ± 12.6	80.8 ± 13.3^†^	83.6 ± 14.7^†#^	< 0.001^*^
BMI (kg/m^2^)	27.0 ± 4.2	28.3 ± 4.1^†^	29.0 ± 4.0^†^	< 0.001^*^
Obesity, n (%)	79 (38.3)	99 (48.5)	117 (56.3)	0.001^*^
Alcohol consumption, n (%)	40 (19.4)	48 (23.5)	54 (26.0)	0.278
Cigarette smoking, n (%)	63 (30.6)	83 (40.7)	99 (47.6)	0.002^*^
Hypertension, n(%)	155 (75.2)	161 (78.9)	175 (84.1)	0.079
Diabetes mellitus, n (%)	51 (24.8)	71 (34.8)	68 (32.7)	0.067
CAD, n (%)	99 (48.1)	96 (47.1)	92 (44.2)	0.720
Antihyperlipidemic medications, n (%)	59 (28.6)	51 (25.0)	41 (19.7)	0.104
≥ 3 classes of anti-hypertensive medications, n (%)	51 (24.8)	56 (27.5)	79 (38.0)	0.008^*^
Hypertension severity				
Stage-2 hypertension, n (%)	72 (35.0)	86 (42.2)	107 (51.4)	0.003^*^
Laboratory parameters				
FBG (mmol/L)	5.41 (4.92 - 6.20)	5.76 (5.21 - 7.28)^†^	5.91 (5.20 - 7.30)^†^	< 0.001^*^
TG (mmol/L)	0.94 (0.74 - 1.20)	1.53 (1.31 - 1.78)^†^	2.78 (2.18 - 3.63)^†#^	< 0.001^*^
TC (mmol/L)	4.21 ± 1.08	4.48 ± 1.11^†^	4.62 ± 1.06^†^	0.001^*^
HDL-C (mmol/L)	1.22 ± 0.26	1.02 ± 0.19^†^	0.89 ± 0.18^†#^	< 0.001^*^
LDL-C (mmol/L)	2.40 ± 0.81	2.69 ± 0.83^†^	2.72 ± 0.71^†^	< 0.001^*^
AIP	-0.10 ± 0.14	0.19 ± 0.07^†^	0.52 ± 0.16^†#^	< 0.001^*^
OCST parameters				
AHI (events/h)	16.7 (10.2 - 30.5)	18.9 (10.2 - 37.2)	19.6 (10.8 - 37.9)	0.210
Mean SpO_2_ (%)	94 (93 - 95)	94 (93 - 95)	94 (93 - 95)	0.253
LSpO_2_ (%)	80 (74 - 83)	79 (74 - 83)	79 (73 - 82)	0.287
TS90 (%)	21 (7 - 51)	26 (8 - 72)	31 (8 - 68)	0.162
OSA severity				0.385
Mild OSA	91 (44.2)	83 (40.7)	78 (37.5)	
Moderate to severe OSA	115 (55.8)	121 (59.3)	130 (62.5)	
Echocardiographic parameters				
LVIDd (mm)	47 ± 4	48 ± 4	48 ± 4^†^	0.026^*^
PWTd (mm)	10 (9 - 10)	10 (9 - 10)	10 (9 - 11)^†#^	0.001^*^
IVSTd (mm)	10 (9 - 11)	10 (10 - 11)	11 (10 - 12)^†#^	< 0.001^*^
LAD (mm)	37 ± 4	37 ± 6	37 ± 4	0.620
LVEF (%)	65 ± 6	64 ± 5	64 ± 6	0.167
LVM (g)	170.4 ± 40.2	177.6 ± 39.0	191.2 ± 52.3^†#^	< 0.001^*^
LVMI (g/m^2^)	92.4 ± 19.4	93.1 ± 19.1	98.2 ± 23.8^†#^	0.009^*^
LVMI_h2.7_ (g/m^2.7^)	42.8 ± 10.5	43.2 ± 9.3	46.1 ± 12.1^†#^	0.003^*^
RWT	0.41 ± 0.05	0.40 ± 0.05	0.42 ± 0.06^†#^	0.037^*^
LVH (%)	52 (25.2)	49 (24.0)	72 (34.6)	0.032^*^
LV geometric pattern				0.053
Normal	109 (52.9)	105 (51.5)	93 (44.7)	
Concentric remodeling	45 (21.8)	50 (24.5)	43 (20.7)	
Eccentric hypertrophy	30 (14.6)	29 (14.2)	30 (14.4)	
Concentric hypertrophy	22 (10.7)	20 (9.8)	42 (20.2)	

*Abbreviations*: *AIP* atherogenic index of plasma, *SBP* systolic blood pressure, *DBP* diastolic blood pressure, *BMI* body mass index, *CAD* coronary artery disease, *FBG* fasting blood glucose, *TG* triglyceride, *TC* total cholesterol, *HDL-C* high-density lipoprotein cholesterol, *LDL-C* low-density lipoprotein cholesterol, *OCST* complete out of center sleep test, *AHI* apnea–hypopnea index, *SpO*_*2*_ oxygen saturation, *LSpO*_*2*_ lowest pulse oxygen saturation, *TS90* the percentage of sleep duration with oxygen saturation < 90%, *OSA* obstructive sleep apnea, *LVIDd* diastolic left ventricular inner diameter, *PWTd* diastolic left ventricular posterior wall thickness, *IVSd* diastolic interventricular septal thickness, *LAD* left atrium diameter, *LVEF* left ventricular ejection fraction, *LVM* left ventricular mass, *LVMI* left ventricular mass index, *LVMI*_*h2.7*_ dividing LVM by height^2.7^, *RWT* relative wall thickness, *LVH* left ventricular hypertrophy, *LV* left ventricular

^*^P value < 0.05

^†^ vs. T1, *P* value < 0.05

^#^ vs. T2, *P* value < 0.05

### Associations between AIP and LV geometric indicators

AIP was positively correlated with LV geometric indicators including the LVMI_h2.7_, LVMI, LVM, LVIDd, PWTd and IVSd (*P* < 0.05, Table 4), according to the correlation analysis. Then, linear regression models were constructed to study the correlation between AIP and LVMI_h2.7_. The results suggested that AIP was significantly associated with LVMI_h2.7_ both in the unadjusted model (β = 0.132, *P* = 0.001) and in the model fully adjusted for age, sex, BMI, SBP, diabetes mellitus, CAD, cigarette smoking, alcohol consumption, antihyperlipidemic medications and AHI (β = 0.125, *P* = 0.001).

**Table 4:** Correlations between AIP and LV geometric indicators

Variables	AIP	
	r	*P* value
LVIDd (mm)	0.128	0.001^*^
PWTd (mm)	0.168	< 0.001^*^
IVSTd (mm)	0.177	< 0.001^*^
LAD (mm)	0.052	0.195
LVEF (%)	-0.069	0.089
LVM (g)	0.221	< 0.001^*^
LVMI (g/m^2^)	0.132	0.001^*^
LVMI_h2.7_ (g/m^2.7^)	0.132	0.001^*^
RWT	0.079	0.051

*Abbreviations*: *LV* left ventricular, *AIP* atherogenic index of plasma, *LVIDd* diastolic left ventricular inner diameter, *PWTd* diastolic left ventricular posterior wall thickness, *IVSd* diastolic interventricular septal thickness, *LAD* left atrium diameter, *LVEF* left ventricular ejection fraction, *LVM* left ventricular mass, *LVMI* left ventricular mass index, *LVMI*_*h2.7*_ dividing LVM by height^2.7^, *RWT* relative wall thickness

^*^P value < 0.05

### Associations between AIP and LVH

**Fig. 3:**
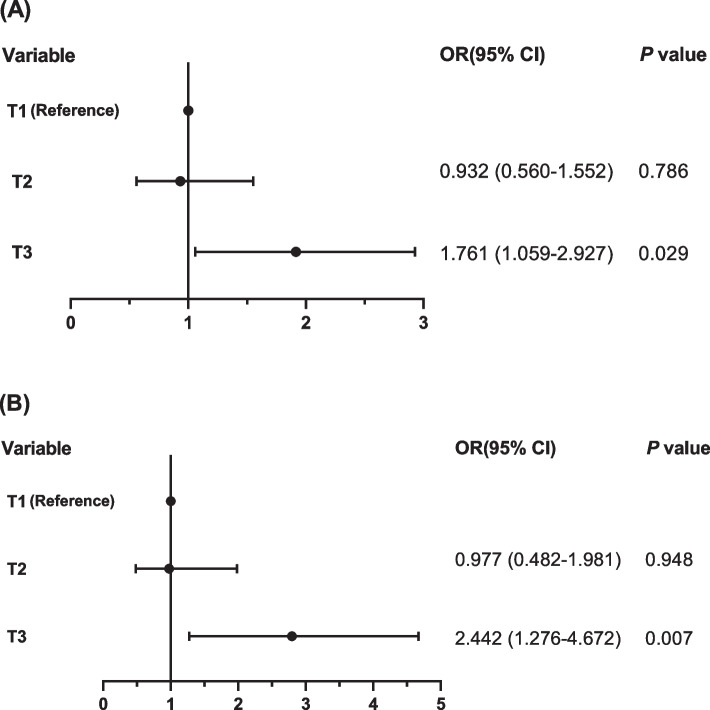
Forest plots of the risk of LVH (**A**) and concentric LVH (**B**) according to tertiles of AIP by the fully adjusted logistic regression model. LVH, left ventricular hypertrophy; AIP, atherogenic index of plasma; BMI, body mass index; OSA, obstructive sleep apnea; OR, odds ratio; CI, confidence interval; T1, AIP < 0.07; T2, 0.07 ≤ AIP < 0.31; T3, AIP ≥ 0.31

The correlation between AIP and LVH was assessed by logistic regression models (Table 5). Univariate logistic regression analysis revealed that AIP was associated with an increased risk of LVH (OR = 1.226 per 1 SD increment, 95% CI: 1.026 - 1.465, *P* = 0.025). In addition, AIP remained independently associated with an elevated risk of LVH (OR = 1.317 per 1 SD increment, 95% CI: 1.058 - 1.639, *P* = 0.014) after fully adjusting all confounding risk factors, including age, sex, BMI, cigarette smoking, alcohol consumption, diabetes mellitus, CAD, hypertension severity, OSA severity and antihyperlipidemic medications. Taking AIP as a categorical variable, the incidence of LVH increased significantly in the T3 group compared to those in the reference T1 group (OR = 1.761, 95% CI: 1.059 - 2.927, *P* = 0.029, Fig. 3A) after fully adjusting for all confounding risk factors.

**Table 5:** Univariate and multivariate logistic regression analyses for analysing the association between AIP and the risk of LVH and concentric LVH

Variables	Univariate		Multivariate	
	OR (95% CI)	*P* value	OR (95% CI)	*P* value
LVH				
AIP (Per 1 SD increase)	1.226 (1.026 - 1.465)	0.025	1.317 (1.058 - 1.639)	0.014^*^
Tertiles of AIP		0.033		0.019^†^
T1 (< 0.07)	1.000 (reference)		1.000 (reference)	
T2 (0.07 - 0.30)	0.936 (0.597 - 1.467)	0.774	0.932 (0.560 - 1.552)	0.786
T3 (≥ 0.31)	1.568 (1.025 - 2.398)	0.038	1.761 (1.059 - 2.927)	0.029
Concentric LVH				
AIP (Per 1 SD increase)	1.438 (1.139 - 1.815)	0.002	1.545 (1.173 - 2.035)	0.002^#^
Tertiles of AIP		0.004		0.003^§^
T1 (< 0.07)	1.000 (reference)		1.000 (reference)	
T2 (0.07 - 0.30)	0.909 (0.480 - 1.723)	0.770	0.977 (0.482 - 1.981)	0.948
T3 (≥ 0.31)	2.116 (1.213 - 3.693)	0.008	2.442 (1.276 - 4.672)	0.007

*Abbreviations*: *AIP* atherogenic index of plasma, *LVH* left ventricular hypertrophy, *OR* odds ratio, *CI* confidence interval, *SD* standard deviation

^*^Hosmer-Lemeshow test’s Chi-square value = 10.093, *P* value = 0.259

^†^Hosmer-Lemeshow test’s Chi-square value = 6.129, *P* value = 0.633

^#^Hosmer-Lemeshow test’s Chi-square value = 12.522, *P* value = 0.129

^§^Hosmer-Lemeshow test’s Chi-square value = 14.831, *P* value = 0.063

### Associations between AIP and concentric LVH

Moreover, AIP remained independently associated with an elevated risk of concentric LVH according to both univariate (OR = 1.438 per 1 SD increment, 95% CI: 1.139 - 1.815, *P* = 0.002, Table 5) and multivariate (OR = 1.545 per 1 SD increment, 95% CI: 1.173 - 2.035, *P* = 0.002, Table 5) models after fully adjusting all confounding risk factors, including age, sex, BMI, cigarette smoking, alcohol consumption, diabetes mellitus, CAD, hypertension severity, OSA severity and antihyperlipidemic medications. In addition, the incidence of concentric LVH increased significantly in the T3 group compared to those in the reference T1 group (OR = 2.442, 95% CI: 1.276 - 4.672, *P* = 0.007, Fig. 3B) after fully adjusting for all confounding risk factors.

## Discussion

This is the first study to explore the correlation between AIP and LV geometric remodeling in OSA patients. The results showed that AIP was significantly higher in OSA patients with LVH and concentric LVH. In addition, the level of LVMI_h2.7_ and the incidence of LVH and concentric LVH were greater in patients with the highest AIP tertile compared with those with the low and middle AIP tertiles. AIP increased along with LVMI_h2.7_. Notably, AIP was independently associated with an increased risk of LVH and concentric LVH after fully adjusting for all confounding risk factors, suggesting that AIP, which is easily measured in routine clinical examinations, could be used as a promising parameter to supplement and improve LVH and concentric LVH detection for OSA patients in clinical practice.

Relevant studies have reported a positive correlation between OSA and LVH. LVM and LVMI were found to be positively and independently correlated with OSA severity [28, 29]. The present study revealed that in patients with OSA, those in LVH group and concentric LVH group had higher levels of AHI and higher prevalence of moderate to severe OSA, which is largely consistent with previously published results. Moreover, a recent meta-analysis demonstrated that AIP was significantly higher in patients with OSA and increased with the severity of OSA [30]. Largely in line with those results, the level of AHI and the prevalence of moderate to severe OSA in the present study was increased gradually from the lowest AIP tertile group to the highest AIP tertile group, although there was no significant statistical difference due to the relatively small sample size.

IR is defined as an impaired glucose-lowering response to insulin stimulation involving the suppression of endogenous glucose production, lipolysis, cellular uptake of available plasma glucose, and net glycogen synthesis, which in turn stimulates insulin secretion to compensate [31]. Insulin directly induces the hypertrophy of cardiomyocytes by increasing protein synthesis and stimulating oxidative stress and inflammation [6, 7, 32]. Moreover, hyperinsulinemia stimulates sympathetic nervous system activity, which may in turn aggravate myocardial hypertrophy [33]. Previous studies have demonstrated an independent association between the degree of IR and increased LVMI and LV mass to volume ratio [34, 35]. In addition, a higher level of serum insulin at baseline and a greater increase in insulin during follow-up were found to independently predict an increase in LVMI [36]. The present study revealed that in the setting of OSA, patients with LVH and concentric LVH were more likely to have adverse cardiometabolic risk factors related to IR, such as older age, higher SBP, BMI and TG levels, and a greater incidence of obesity and stage-2 hypertension. Given that IR is an important mechanism of LVH and that OSA has been demonstrated to be a risk factor for IR [37–40], these results further emphasized that IR might be one of the underlying mechanisms of LVH in OSA patients.

In recent years, easily measured and cost-effective surrogate indicators of IR, such as triglyceride and glucose (TyG) index and AIP, have received widespread attention in the field of cardiometabolic multimorbidity detection and management [41]. Studies have shown that TyG index and AIP were independently associated with IR-related metabolic diseases, such as CAD, metabolic syndrome and hypertension [11]. In addition, AIP was demonstrated to be more strongly associated with metabolic syndrome than TyG index in Spanish adolescents [42]. In the presence of IR, TG levels are often increased, while HDL-C levels are often decreased [43, 44]. Therefore, AIP, determined as log_10_ (TG/ HDL-C), was supposed to have good correlation with IR. A large cross-sectional study involving 9,245 US adults showed that AIP was positively associated with homeostasis model assessment of IR (HOMA-IR), the gold standard method to measure IR, after adjusting for potential confounding variables both in patients with and without diabetes [8]. Although the correlation of AIP with IR has not been evaluated in the current study, the results that patients with high AIP values had important anthropometric features of IR, such as higher DBP and BMI and a greater incidence of obesity, smoking and stage-2 hypertension, further indicated the good correlation of AIP with IR-related metabolic diseases.

Few studies have explored the relationship between AIP and LV geometric remodeling. In childhood, a high TG/HDL-C ratio was found to be associated with an increased concentric LVH risk, especially in obese children. In an outpatient population of white children and adolescents in Italy, Procolo and his colleagues first demonstrated that LVMI increased across tertiles of the TG/HDL-C ratio, and children with a high TG/HDL-C ratio (> 2.0) showed a 2.62-fold greater risk of concentric LVH [17]. Similar results were found three years later by the authors in a larger population of 5055 overweight and obese children [18]. In another two recent cross-sectional studies conducted in Serbia and Turkey, a positive association between TG/HDL-C ratio and LVMI was found in obese children [19, 20]. In adults, AIP was found to positively correlate with LVMI in morbid obesity adults without underlying cardiac diseases [45]. However, the positive association was not statistically significant when adjusted for confounding factors. Interestingly, Haoyu Wang et al*.* analysed a large population of 10,756 participants of rural China and reported that TG/HDL-C ratio was positively and independently correlated with both LVMI and increased concentric LVH risk according to the multivariable adjusted regression models [21].

Extending those prior studies, the present study further explored the association between AIP and LV geometric remodeling in adult OSA patients and found that AIP was positively correlated with LV geometric indicators, including LVIDd, PWTd, IVSd, LVM and LVMI. The positive association between AIP and LVMI was still significant according to multivariable adjusted linear regression models, which was in line with the findings of Haoyu Wang et al. in the general population [21]. Furthermore, AIP was strongly related to the prevalence of LVH and concentric LVH beyond traditional risk factors, including age, BMI and hypertension severity. Combined with the results of previous studies in children and general population, the current findings in OSA patients further demonstrated the close correlation of AIP with LVH risk, suggesting that AIP might be used as an available marker to monitor hypertrophic remodeling of the heart and improve CVDs risk stratification in the clinical management of OSA. However, considering the relatively small sample size of this study, studies with larger populations are required to identify an accurate reference value of AIP for clinical guidance.

The present study revealed an independent association of high AIP with increased LVH and concentric LVH risk in OSA patients but did not provide a direct mechanism to explain these results. However, some data from the literature may be helpful. First, HDL-C has been proven to exert direct inhibitory effects on myocardial hypertrophy, including inhibiting cardiomyocyte hypertrophy in vitro and cardiac hypertrophy in vivo, exerting pro-survival effects on endothelium, and directly inhibiting cardiac fibrosis [46]. Second, although TG stored within lipid droplets has no direct toxic effect on the myocardium, a recent study in spontaneously hypertensive rats (SHRs) showed that the progression of LVH and heart dysfunction in SHRs was associated with TG accumulation, which was related to an increase in the expression of genes involved in TG synthesis and a decrease in the rate of lipolysis and β-oxidation of fatty acids in cardiomyocytes [47]. Collectively, in consideration of the prohypertrophic property of TG and the antihypertrophic property of HDL-C, AIP may have a prohypertrophic impact on the myocardium. The present finding of an independent association of high AIP with increased risk of LVH and concentric LVH supports the hypothesis that in patients with OSA, IR-related dyslipidemia is one of the underlying pathophysiologic bases of cardiac hypertrophic remodeling.

### Strengths and limitations

The strength of the current study lies in the fact that it is the first study to explore the association between AIP and LV geometric abnormalities in OSA patients. The results demonstrated a close and positive relationship between AIP and LV geometric indicators and an independent and positive relationship between increased AIP and elevated risk of LVH and concentric LVH in OSA patients.

Meanwhile, some limitations can be envisaged. First, the retrospective cross-sectional nature of this single center study precludes us from establishing a definite causal relationship between AIP and LVH. However, the present findings highlight the importance of AIP in OSA patients at risk of LVH and concentric LVH. Second, the sample size of this study was relatively small, and all of the participants were of Chinese ethnicity, which means that the findings may not be generalizable to other ethnic groups. Third, the gold standard IR parameters, such as HOMA-IR, were not analysed in this study due to a lack of data. However, a close relationship between AIP and HOMA-IR has been demonstrated in previous studies.

## Conclusion

In OSA patients, AIP increased along with LVMI and was independently associated with an increased risk of LVH and concentric LVH regardless of age, BMI or hypertension severity. As LVH and concentric LVH are independent risk factors for cardiovascular events, AIP, a practical and cost-effective test, could be included in the routine examination of OSA patients, so as to help identify the high-risk population of LVH and concentric LVH and reduce consequent cardiovascular morbidities in the clinical management of OSA.

## Data Availability

The datasets used and analysed during the current study are available from the corresponding author upon reasonable request.

## Abbreviations

OSA: Obstructive sleep apnea
LVH: Left ventricular hypertrophy
CVDs: Cardiovascular diseases
IR: Insulin resistance
AIP: Atherogenic index of plasma
TG: Triglyceride
HDL-C: High-density lipoprotein cholesterol
CAD: Coronary artery disease
LVMI: Left ventricular mass index
AHI: Apnea-hypopnea index
OCST: Complete out of center sleep test
SBP: Systolic blood pressure
DBP: Diastolic blood pressure
BMI: Body mass index
BP: Blood pressure
FBG: Fasting blood glucose
TC: Total cholesterol
LDL-C: Low-density lipoprotein cholesterol
TS90: The percentage of sleep duration with oxygen saturation < 90%
LSpO_2_: Lowest pulse oxygen saturation
SpO_2_: Oxygen saturation
LAD: Left atrium diameter
LVIDd: Diastolic left ventricular inner diameter
PWTd: Diastolic left ventricular posterior wall thickness
IVSTd: Diastolic interventricular septal thickness
LVEF: Left ventricular ejection fraction
LVM: Left ventricular mass
BSA: Body surface area
RWT: Relative wall thickness
SD: Standard deviation
ANOVA: One-way analysis of variance
LV: Left ventricular
OR: Odds ratio
CI: Confidence interval
TyG: Triglyceride and glucose
HOMA-IR: Homeostasis model assessment of IR
SHRs: Spontaneously hypertensive rats
